# Cigarette smoking promotes bladder cancer via increased platelet‐activating factor

**DOI:** 10.14814/phy2.13981

**Published:** 2019-02-12

**Authors:** Shannon Kispert, John Marentette, Jane McHowat

**Affiliations:** ^1^ Department of Biology University of North Georgia Oakwood Georgia; ^2^ Skaggs School of Pharmacy and Pharmaceutical Sciences University of Colorado Anschutz Medical Campus Aurora Colorado; ^3^ Department of Pathology Saint Louis University School of Medicine St. Louis Missouri

**Keywords:** Bladder cancer, cigarette smoke, platelet activating factor

## Abstract

Cigarette smoking is the number one risk factor for bladder cancer development and epidemiological data suggest that nearly half of all bladder cancer patients have a history of smoking. In addition to stimulating the growth of a primary tumor, it has been shown that there is a correlation between smoking and tumor metastasis. Platelet activating factor (PAF) is expressed on the cell surface of the activated endothelium and, through binding with the PAF‐receptor (PAF‐R), facilitates transendothelial migration of cells in the circulation (McHowat et al. *Biochemistry* 40:14921–14931; 2001). In this study, we show that the exposure of bladder cancer cells to cigarette smoke extract (CSE) results in increased PAF accumulation and increased expression of the PAF‐R. Furthermore, treatment with CSE increases adherence of bladder cancer cells to bladder endothelial cells and could be abrogated by pretreatment with ginkgolide B. Immunohistochemical analysis of tumor biopsy samples from bladder cancer patients who smoked revealed increased PAF and the PAF‐R in tumor regions when compared to normal tissue. These data highlight a pathway in bladder cancer that is influenced by CSE which could facilitate primary tumor growth and increase metastatic potential. Targeting of the PAF‐PAFR interaction could serve as a beneficial therapeutic target for managing further growth of a developing tumor.

## Introduction

Bladder cancer remains a major global health burden. In the United States alone, there are nearly 80,000 new cases estimated for 2017 (SEER Cancer Statistics Review, 2017). Bladder cancer is the sixth most common cancer and the mortality rate remains high with an estimated 16,870 deaths this year alone (SEER Cancer Statistics Review, 2017). Currently in the United States there are more than half a million people estimated to be living with bladder cancer (SEER Cancer Statistics Review, 2017). Signs and symptoms of bladder cancer include blood in the urine, frequent urination, and pain during urination (PDQ® Adult Treatment Editorial Board, 2017). Risk factors for bladder cancer include family history, history of bladder infections, previous radiation therapy, and cigarette smoking (PDQ® Adult Treatment Editorial Board, 2017).

Nearly 17% of U.S. adults continue to smoke despite direct evidence that cigarette smoking causes numerous types of cancers and is the leading cause of death and preventable disease (Department of Health and Human Services, Public Health Service, Office of the Surgeon General, 2014; Jamal et al. 2015). Tobacco smoking remains the best‐established risk factor for bladder cancer to date (International Agency for Research on Cancer, 2004). In recent studies, we have discovered that smoking is associated with increased platelet‐activating factor (PAF) accumulation and PAF receptor (PAF‐R) expression and may contribute to tumor progression and metastasis in smokers (Kispert et al. 2014, 2015, 2017a,b). PAF has a well described role in inflammatory cell recruitment to the endothelium during inflammation, and has also been implicated in primary tumor growth and metastasis in several published studies (Im et al. 1996; Camussi et al. 1996; Camussi et al. 1997; Montrucchio et al. 1998, 2000; Bussolati et al. 2000; Denizot et al. 2001; Melnikova et al. 2006; Melnikova and Bar‐Eli 2007; McHowat et al. 2011). PAF is generated via hydrolysis of membrane phospholipids, mediated by calcium‐independent phospholipase A_2_ (iPLA_2_) which removes the *sn*‐2 fatty acid yielding a free fatty acid, arachidonic acid, and a lysophospholipid which can act as the precursor for PAF production (McHowat et al. 2001). Under physiological conditions, PAF is maintained at low concentrations by PAF‐acetylhydrolase (PAF‐AH) which removes the sn‐2 acetate group from PAF, resulting in biologically inactive lyso‐PAF.

We have previously shown that exposure of human urothelial cells (HUC) to cigarette smoke extract (CSE) stimulates the production of PAF, inhibits its hydrolysis, and increases PAF‐R expression (Kispert et al. 2017b). Exposure of mice to long‐term cigarette smoking revealed disruption of urothelial integrity and increased accumulation of PAF in the bladder wall (Kispert et al. 2017b). In this study, we have examined the role of cigarette smoke on PAF and PAF‐R in bladder cancer cells. In addition, we have studied the effects of inhibition of iPLA_2_ *β*, the predominant isoform responsible for PAF production, on CSE‐mediated PAF accumulation in bladder cancer cells, and studied the effect of the PAF‐R blocker ginkgolide B on adherence of bladder cancer cells to the bladder endothelium. These latter studies highlight the therapeutic potential of targeting PAF accumulation and interaction with its receptor in tumor management. To affirm these findings, we have performed preliminary studies in smokers with bladder cancer to determine PAF accumulation and PAF‐R expression.

## Methods and Materials

### Cell culture

Primary human urothelial cells (HUC) were obtained from ScienCell Research Laboratories (Carlsbad, CA). Urothelial cell cultures were grown in EpiLife Media (Cascade Biologics, Inc. Portland, OR) with calcium (0.06 mmol/L), growth factor supplements provided by the manufacturer and penicillin (20 U/mL)/streptomycin (100 mg/mL) and incubated at 37°C, with an atmosphere of 95% O_2,_ 5% CO_2_. Confluent monolayers were differentiated by adding 1 mmol/L calcium and 10% fetal bovine serum (Ca/FBS). Experiments were performed after 3 days of differentiation. Human grade II urinary bladder carcinoma cells, HTB‐9 (ATCC, Manassas, VA), were grown in RPMI‐1640 medium, supplemented with 10% fetal bovine serum and penicillin (20 U/mL) and streptomycin (100 mg/mL). Human grade III urinary bladder carcinoma cells, HT‐1376 (ATCC, Manassas, VA), were grown in Eagle's Minimum Essential Medium supplemented with 10% fetal bovine serum and penicillin (20 U/mL) and streptomycin (100 mg/mL). Bladder cancer cells were used when confluent. Human bladder microvascular endothelial cells (HBMEC) were grown in EGM‐2MV medium (Lonza, Walkersville, MD) and maintained at 37°C in a humidified atmosphere of 95% O_2_ and 5% CO_2_. Cells were treated with cigarette smoke extract (CSE, 20 *μ*g/mL) for indicated times either with or without pretreatment with ginkgolide B (10 *μ*mol/L, 30 min) or (S)‐bromoenol lactone (5 *μ*mol/L, 30 min). CSE was obtained from Murty Pharmaceuticals (Lexington, KY). (S)‐bromoenol lactone and ginkgolide B were obtained from Cayman Chemical Co. (Ann Arbor, MI). All other materials were obtained from Sigma Chemical Company (St. Louis, MO).

### Cell adherence to bladder endothelium

HTB‐9 or HT‐1376 cells were labeled with calcein‐AM (4 *μ*g/mL, Alexis Biochemicals, Lausen, Switzerland) for 45 min at 37°C. After washing three times, 2 × 10^6^ cells were layered onto confluent bladder endothelial cell monolayers. Medium and unbound cells were removed and discarded. Adherent bladder cancer cells and endothelial cells were washed with Dulbecco's phosphate buffered saline and lysed with 1 mL of 0.2% Triton X‐100. Samples were sonicated (550 Sonic Dismembrator, Fisher Scientific, Pittsburgh, PA) for 10 sec. The amount of calcein‐AM fluorescence was measured using a Synergy 2 microplate reader at an excitation wavelength of 485 nm and emission wavelength of 530 nm. The percent of cell adherence was calculated from the amount of calcein‐AM fluorescence measured in 2 × 10^6^ cells.

### PAF production

Human urothelial and bladder cancer cells grown to confluence were incubated with Hanks’ balanced salt solution containing 10 *μ*Ci of [^3^H] acetic acid for 20 min at room temperature. After experimental conditions, cell lipids were extracted using the Bligh and Dyer method (Bligh and Dyer 1959). Total lipid extracts were resuspended in 9:1 CHCl_3_:MeOH and applied to TLC plates. Plates were developed in 100:50:16:8 chloroform, methanol, acetic acid, and water. The region corresponding to [^3^H] PAF was scraped and measured by liquid scintillation counting.

### Immunoblot analysis

Bladder cells were suspended in lysis buffer containing (mmol/L) HEPES 20 (pH 7.6), sucrose 250, dithiothreitol 2, EDTA 2, EGTA 2, *β*‐glycerophosphate 10, sodium orthovanadate 1, phenylmethylsulfonyl fluoride 2, leupeptin 20 *μ*g/mL, aprotinin 10 *μ*g/mL, and pepstatin A 5 *μ*g/mL. Cells were sonicated on ice and centrifuged at 20,000*g* at 4°C for 20 min to remove cellular debris and nuclei. Cytosolic protein was separated by SDS/PAGE and electrophoretically transferred to nitrocellulose membranes (Bio‐Rad, Richmond, CA). The blocked nitrocellulose membrane was incubated with primary antibody (anti‐PAF receptor, 1 in 1000 dilution, Cayman Chemical Co., Ann Arbor, MI) and horseradish peroxidase‐conjugated secondary antibody (anti‐rabbit, 1 in 10,000 dilution, Fisher Scientific). Regions of antibody‐binding were detected using enhanced chemiluminescence (Amersham, Arlington Heights, IL) after exposure to film (Hyperfilm, Amersham). Equal loading was verified by immunoblot analysis for *β*‐actin.

### Immunohistochemistry

Normal and tumor bladder tissue was obtained from patients treated at Saint Louis University Hospital. All bladder cancer patients were current or former smokers, with former smokers having at least a 25‐pack year history. Bladder tumors were classified as low‐grade or high‐grade by a pathologist. Bladder cancer tissue for immunohistochemistry was fixed in 10% buffered formalin, embedded in paraffin, and cut into 5 *μ*m thick sections. Tissue was deparaffinized and rehydrated in xylene and decreasing concentrations of reagent alcohol. Sections underwent heat‐induced antigen retrieval using a citric acid‐based antigen unmasking solution (Vector Labs). Sections were incubated with blocking buffer and PAF (Abbiotec, San Diego, CA) or PAF‐R (Cayman Chemical Company, Ann Arbor, MI) primary antibody overnight. Immunohistochemistry was completed with the use of horseradish peroxidase‐conjugated secondary antibodies (Jackson Labs) and diaminobenzidine tetrahydrochloride (DAB, Biogenex). Slides were counterstained with filtered Gills III hematoxylin and blued in saturated lithium carbonate solution and viewed under light microscope. Immunohistochemistry was quantified using image analysis by ImageJ FIJI wherein % area of DAB positive signal was calculated. *n* ≥ 3 patients per group and *n* ≥ 3 microscopic fields per patient for quantification. All immunohistochemistry with patient experimental samples as well as staining controls were performed concurrently for uniformity of results.

### Statistical methods

Data were analyzed using Student's *t*‐test and are means + SEM. Differences were regarded as significant at *P* < 0.05 and *P* < 0.01.

## Results

### In vitro studies using human urothelial and bladder cancer cells

**Figure 1:**
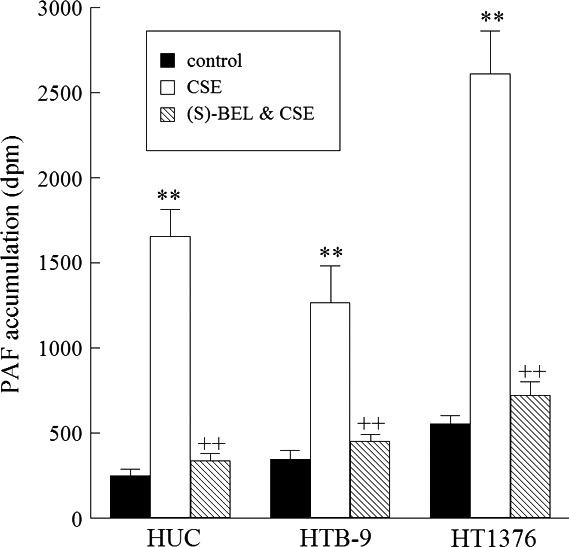
CSE incubation results in significant PAF production which can be blocked by pretreatment with (S)‐BEL to inhibit iPLA_2_ *β* activity Platelet‐activating factor (PAF) accumulation in human urothelial cells (HUC), and bladder cancer cell lines HTB‐9 and HT1476 incubated with media alone (black boxes), CSE (20 *μ*g/mL, 48 h, white boxes), or CSE following pretreatment with (S)‐BEL (5 *μ*mol/L, 30 min, checked boxes). Values shown are means + SEM for four separate cultures. **P < 0.01 when compared to controls. ^++^ *P* < 0.01 when comparing in the presence and absence of inhibitor.

We have previously shown that cigarette smoking increases PAF production in breast cancer cells as well as bladder cells from patients with interstitial cystitis/painful bladder syndrome (Kispert et al. 2015, 2017b). To determine whether similar changes were observed in bladder cancer, we performed similar studies using bladder urothelial cells. HTB‐9 cells, a human bladder cancer grade II line, and a more aggressive grade III bladder cancer cell line, HT‐1376, were selected to represent low‐grade and high‐grade bladder tumors respectively. For comparison to normal urothelium, we utilized primary human urothelial cells (HUC) isolated from the urinary bladder. HUC, HTB‐9, and HT‐1376 cells were incubated with CSE for 48 h and PAF accumulation measured (Fig. 1). Following incubation, HUC, HTB‐9, and HT‐1376 all exhibited significant increases in PAF accumulation, with the greatest increase observed in grade III HT‐1376 cells (Fig. 1). We have demonstrated previously that the majority of PLA_2_ activity that is responsible for PAF production is calcium independent PLA_2_ *β* (iPLA_2_ *β*) (Rastogi and McHowat 2009; Sharma et al. 2010). Cells were pretreated with the iPLA_2_ *β*‐selective inhibitor (S)‐bromoenol lactone ((S)‐BEL) for 30 min prior to incubation with CSE. Our previous studies in endothelial and urothelial cells have demonstrated (*S*)‐BEL pretreatment to have no significant effect on basal PAF content after 30 min incubation (data not shown). However, incubation with (*S*)‐BEL prior to the addition of CSE abrogated CSE‐induced PAF accumulation in all cells studied, indicating that CSE‐induced PAF accumulation is mediated through iPLA_2_ *β* (Fig. 1).

**Figure 2:**
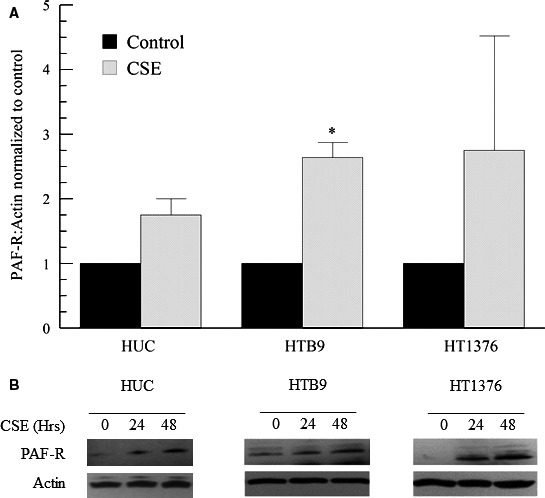
CSE incubation increases PAF‐R expression in human urothelial cells (HUC) and bladder cancer cell lines HTB‐9 and HT1376. Platelet‐activating factor receptor (PAF‐R) expression was analyzed via immunoblot in cells incubated with media alone (black boxes) or CSE (20 *μ*g/mL, 48 h, gray boxes) (A). Values shown are means + SEM for four separate cultures. **P < 0.01 when compared to controls. Representative immunoblots (B).

For PAF to be active it must bind to the PAF‐R. We have investigated the expression of the PAF receptor in bladder cancer cells in response to CSE exposure via immunoblot analysis (Fig. 2B). Following 48 h of CSE exposure, HUC exhibit modest increases in PAF‐R expression when compared to greater than twofold increases in HTB‐9 and HT‐1376 bladder cancer cells (Fig. 2A). This graded response is similar to that observed previously in breast cancer cells and may have similar consequences in tumor progression (Kispert et al. 2014, 2017a).

**Figure 3:**
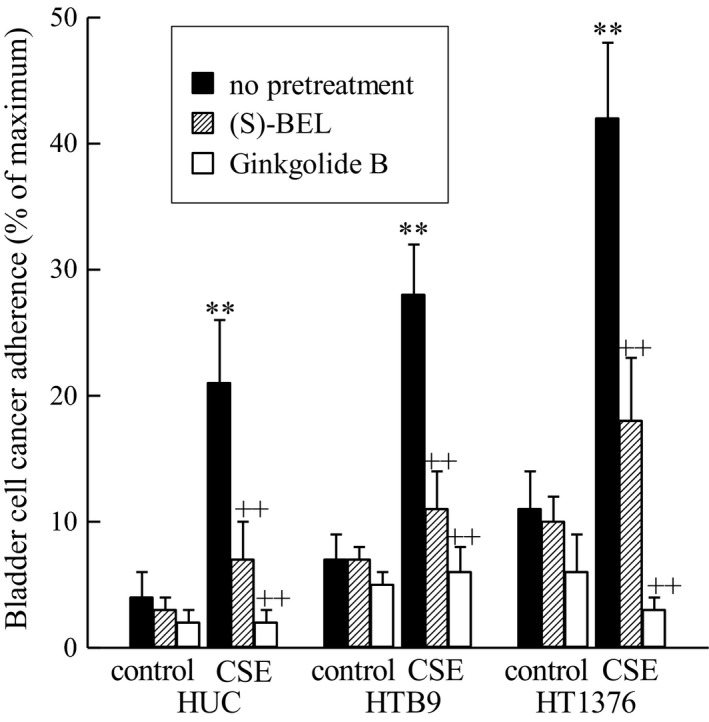
CSE incubation increases adherence of bladder cancer cells to bladder endothelial cells. Human bladder microvascular endothelial cells were incubated with CSE (20 μg/ml, 24 h) prior to incubation with bladder cancer cells. Bladder cancer cells were pretreated with (S)‐BEL (5μM, 30min, checked bars), ginkgolide B (10 μM, 30 min, white bars) or media alone (black bars) and adherence to bladder endothelial cells was measured. **p < 0.01 when compared to controls. ++p < 0.01 when comparing in the presence and absence of inhibitor. n = 4.

To investigate the effects of CSE on bladder cancer invasiveness and potential transendothelial cell migration of tumor cells into the circulation, we incubated human bladder microvascular endothelial cells (HBMEC) with CSE (20 *μ*g/mL, 48 h) and measured adherence of bladder urothelial and tumor cells to the endothelial cell monolayer (Fig. 3). We observed increased cell adherence to bladder endothelial cells following CSE exposure. These effects were similar to those observed previously using breast cancer cells. Pretreatment of HBMEC with (S)‐BEL, the iPLA_2_ *β* inhibitor (5 *μ*mol/L, 30 min prior to CSE addition), diminished the cell adherence in the presence of CSE (Fig. 3). Furthermore, pretreatment of bladder urothelial or tumor cells with ginkgolide B, a PAF‐R antagonist (10 *μ*mol/L, 30 min) prior to addition to HBMEC completely blocked cell adherence (Fig. 3). These data indicated that blocking PAF accumulation or the PAF‐R could be used as a potential therapeutic targets to inhibit bladder tumor cell transmigration across the bladder endothelium.

### Studies using human bladder cancer biopsies

**Figure 4:**
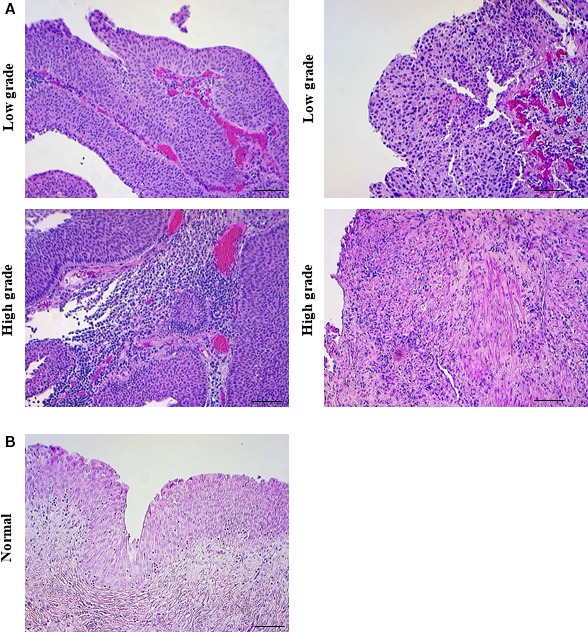
Low‐grade and high‐grade bladder cancer tissue from patients with a history of smoking. Patients with a smoking history were selected for this study and their tissue biopsies were categorized into low‐grade (top panels A, representative images) or high‐grade tumors (bottom panels A, representative images). Non‐diseased bladder tissue (B, representative image). Scale bars: 200 *μ*m.

**Figure 5:**
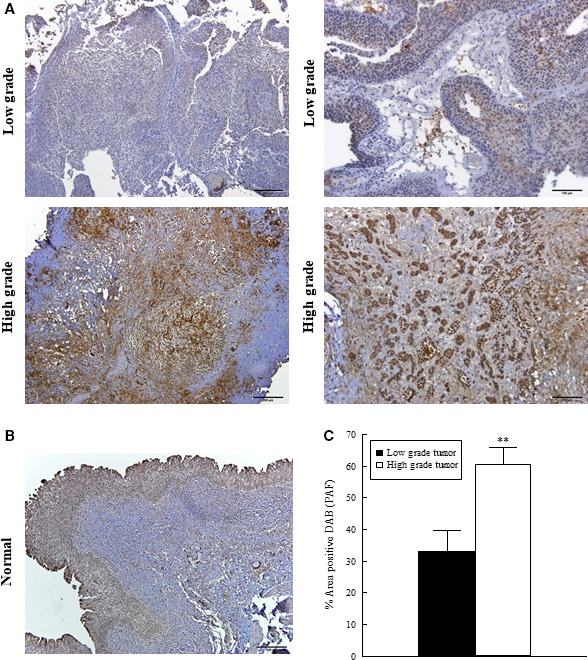
Immunohistochemical expression of PAF in low‐ and high‐grade bladder cancer. Immunohistochemistry for PAF in low‐grade (top panels A, representative images) and high‐grade (lower panels A, representative images) tumors from smoker patients. PAF expression in normal human urothelial tissue (representative image, B). Quantification of PAF signal in tumor tissues (C). Values shown are means + SEM for three separate patient samples. **P < 0.01 when compared to controls. Scale bars: 200 *μ*m.

**Figure 6:**
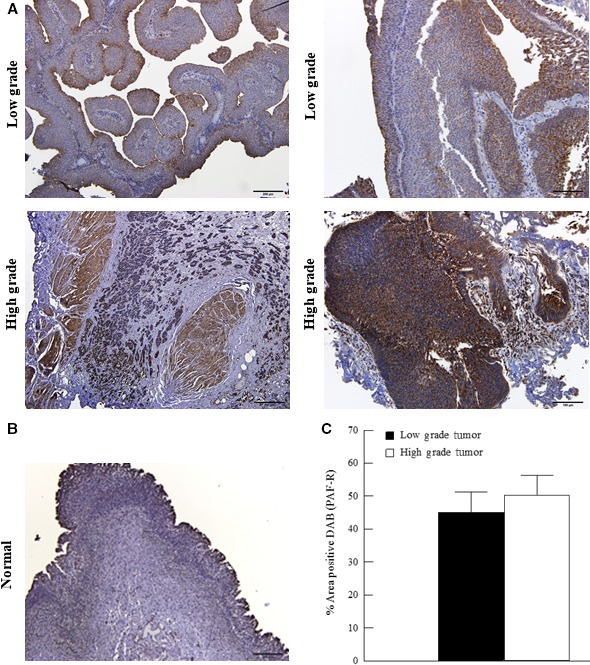
Immunohistochemical expression of PAF‐R in low‐ and high‐grade bladder cancer. Immunohistochemistry for PAF‐R in low‐grade (top panels A, representative images) and high‐grade (lower panels A, representative images) tumors from smoker patients. PAF‐R expression in normal human urothelial tissue (representative image, B). Quantification of PAF‐R signal in tumor tissues (C). Values shown are means + SEM for three separate patient samples. **P < 0.01 when compared to controls. Scale bars: 200 *μ*m.

To investigate the role of PAF and the PAF‐R in bladder cancer, we examined tissue from a small group of patients with bladder cancer (Fig. 4A) and no presence of disease (Fig. 4B). Immunohistochemistry revealed higher expression of PAF (Fig. 5A) when compared to normal urothelium (Fig. 5B). We also compared high‐grade tumors (Fig. 5A lower panel) to low‐grade tumors (Fig. 5A upper panel) and found that PAF expression was significantly elevated in high‐grade tumors (Fig. 5C). When investigating PAF‐R expression, we observed a higher expression of PAF‐R in tumor areas (Fig. 6A) when compared to normal tissues (Fig. 6B). When comparing high‐grade (Fig. 6A lower panel) and low‐grade tumors areas (Fig. 6A upper panel), we detected no significant difference in PAF‐R expression (Fig. 6C.).

**Figure 7:**
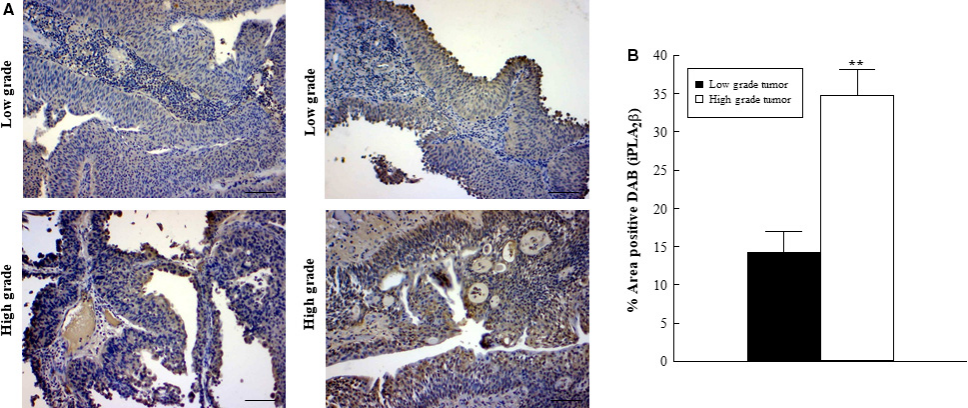
Immunohistochemical expression of iPLA2*β* in low‐ and high‐grade bladder cancer. Immunohistochemistry for iPLA2*β* in low‐grade (top panels A, representative images) and high‐grade (lower panels A, representative images) tumors from smoker patients. Quantification of iPLA2*β* signal in tumor tissues (B). Values shown are means + SEM for four separate patient samples. *^*^ *P* < 0.01 when compared to controls. Scale bars: 200 *μ*m.

We have previously demonstrated iPLA_2_ *β* to be the predominant isoform responsible for PAF production and show here that CSE‐induced PAF accumulation in bladder urothelial and tumor cells can be blocked by pretreatment with (S)‐BEL (Fig 1). In addition, pretreatment of HBMEC with (S)‐BEL inhibits the adherence of bladder urothelial and tumor cells to the endothelium (Fig. 3). To investigate changes in iPLA2*β* expression in human bladder tumor, we examined its expression via immunohistochemistry in our patient samples (Fig 7). Our data revealed a significantly higher expression of iPLA_2_ *β* (Fig 7B) in high‐grade tumors (Fig 7A, lower panel) when compared to low‐grade tumors (Fig 7A, upper panel) from bladder cancer patients, suggesting that iPLA_2_ *β* may play a role in tumor progression and be responsible for the increased PAF expression observed.

## Discussion

Despite immense efforts to eradicate tobacco smoking it still exists as a serious public health concern. Over 17% of all US adults are smokers and it remains the leading preventable cause of death and disease (Agaku et al. 2014). While cancers such as lung and esophageal have been widely studied due to their links with cigarette smoking, much still needs to be elucidated regarding the effects of cigarette smoking on bladder cancer. A recent survey found that 94% of urology patients recognized the associated of smoking and lung cancer, yet only 25% knew the association of bladder cancer with smoking (Bjurlin et al. 2012). Interestingly, tobacco smoking remains the most prominent risk factor for bladder cancer and nearly 50% of bladder cancer cases can be attributed to smoking (Silverman et al. 2006). Recent cohort studies have determined that the relative risk for smoking and bladder has increased in the United States in recent years, perhaps reflecting a change in cigarette composition (Freedman et al. 2011).

PAF is a potent lipid mediator of vascular and inflammatory processes that has more recently been implicated in tumor growth, angiogenesis, and metastasis (Bussolino et al. 1995). PAF was first associated with smoking when it was shown to be elevated in patient plasma following cigarette smoke exposure (Imaizumi 1991). Since that initial study, the role of PAF has been studied in many disease processes that are associated with smoking, but many questions remain unanswered. In this study, we observed significant increases in PAF accumulation in all urothelial and bladder cancer cells following CSE exposure (Fig. 1). Of particular interest, the greatest increases were observed in the most aggressive cell line, grade III HT‐1376 cells. We observed similar responses in breast cancer cells with the most dramatic increases in PAF accumulation in the highest grade tumor cell lines, suggesting that this pathway may be implicated in more aggressive cancers (Kispert et al. 2015). Aggressive cancers are more difficult to treat with a deficit of specific targeted therapies, and are generally associated with a poor prognosis. Considering PAF production is increased in these aggressive bladder cancer cell lines, it may represent a contributing cause for the difficulty in treatment. Agents that target the PAF‐PAF receptor interaction could represent a therapeutic target for the large subset of bladder cancer patients who smoke (Jögi et al. 2012).

We proposed that PAF and its receptor may play a role not only in tumor cell interactions but in the interactions between tumor cells and endothelial cells. A potential therapy for PAF‐mediated maladies include blockade of the PAF‐R, rendering the effects of PAF inactive. We have shown increases in the PAF‐R following CSE incubation in various tumor cell lines including bladder and breast (Fig. 3) (Kispert et al. 2014).

A natural PAF‐R antagonist, ginkgolide B, was used to block the PAF‐R on bladder cancer cells before measuring their adherence to the bladder endothelium (Fig. 3). Without pretreatment, bladder cancer cells showed significant increases in adherence to bladder endothelial cells in response to CSE exposure (Fig. 3). Upon pretreatment with the PAF‐R antagonist ginkgolide B, the adherence was abrogated for both bladder cancer cell lines close to baseline (Fig. 3). These data prove that smoking has a potential effect on tumor progression via cancer cell adherence and transmigration across endothelial cells resulting in cancer cell distribution and metastasis, and highlight the PAF‐R as an important therapeutic target in this pathway. This result is important not only for its novelty in bladder cancer but also because of the similarity to results we have shown in breast cancer and prostate cancer (Kispert et al. 2016). We observed increased adherence of breast cancer cells to lung endothelial cells, a common site of breast cancer metastasis, that was diminished with ginkgolide B treatment (Kispert et al. 2014). Similar results were seen with PC3 prostate cancer cells to lung endothelial cells when incubated with CSE and diminished with ginkgolide B pretreatment (Kispert et al. 2016). All of this work strongly suggests that CSE‐induced cell adherence due to the PAF‐PAF‐R interaction is not specific to one cancer, but may be a general pathway for many cancers.

We proposed this increased adherence following CSE exposure and subsequent PAF/PAF‐R interaction is a result of endothelial cell dysfunction in smokers. As previously mentioned, endothelial cells are a critical component for the establishment of primary tumors and progression to metastatic sites and previous studies have shown that their dysfunction directly contributes to these processes (Franses et al. 2013; Jahroudi et al. 1995). Coincidentally, cigarette smoking is known to establish endothelial cell dysfunction (Messner and Bernhard 2014). The complete abrogation of bladder urothelial or tumor cell adherence by ginkgolide B pretreatment to block the PAF‐R directly implicates the PAF‐PAF‐R interaction as a mechanism for communication and tumor progression between bladder urothelial/cancer cells and bladder endothelial cells. Of importance, these in‐vitro studies have been performed with whole cigarette smoke extract to reflect the typical market cigarette, yet all components may not be able to access the bladder tissue. Future studies in‐vitro should aim at determining which components capable of bladder access are responsible for PAF and PAF‐R changes in these cell lines as a means for identifying future therapeutic targets.

Subsequent to studies performed in bladder cancer cell lines, we determined whether similar changes could be observed in bladder cancer patient tissue. While the sample size is small and major conclusions cannot be drawn, it does provide interesting insight into future directions. Tumor samples were derived from patients with a smoking history and categorized as low‐grade and high‐grade tumors by a pathologist. Immunohistochemistry for iPLA_2_ *β* and PAF revealed increased expression in higher grade tumor regions when compared to low‐grade tumors (Figs. 5 and 7) suggesting that this specific pathway may play a role in tumor progression in the bladder. In contrast, both low‐ and high‐grade tumor areas exhibited high expressions of the PAF‐R with no significant differences between high‐ and low‐grade tumors (Fig. 6). This suggests that any difference in PAF‐mediated effects between tumor grades would likely be mediated via the increase in PAF content rather than an upregulation of the PAF‐R. Although we did not detect a difference in PAF‐R expression between tumor grades, there does appear to be an increase in expression between tumor and adjacent or normal bladder tissue as evidenced in Figure 6. This increased PAF‐R expression in tumor cells may play a role in invasion and metastasis if there is increased cell adherence and transmigration across an activated endothelium that is facilitated by the PAF‐PAF‐R interaction. Although the number of patient samples used in this study is small, we can conclude that PAF and the PAF‐R are consistently detected in the bladder tissue and expression is increased when compared to the adjacent normal urothelial tissue. Unfortunately, biopsies of non‐diseased tissues were difficult to obtain and could not be used for statistical comparison in these studies.

These data highlight a pathway that is upregulated in bladder cancer and that is influenced by CSE which could facilitate primary tumor growth and increase metastatic potential. Targeting of the PAF‐PAFR interaction could serve as a beneficial therapeutic target for managing further growth of a developing tumor. While the PAF and PAF‐R both appear upregulated in response to CSE exposure, future studies will aim at discovering the distinct pathways responsible for each independent outcome. In addition, future studies will be important in elucidating the interaction between bladder endothelial cells and cancer cells as well as verifying this pathway in vivo.

## Conflict of Interest

The authors declare that they have no conflicting interests.
